# Spatially Resolved Microglia/Macrophages in Recurrent Glioblastomas Overexpress Fatty Acid Metabolism and Phagocytic Genes

**DOI:** 10.3390/curroncol31030088

**Published:** 2024-02-23

**Authors:** Akshitkumar M. Mistry, Jonah Daneshmand, SeonYeong Jamie Seo, Norman L. Lehman, Donald M. Miller, Dylan A. Goodin, Hermann B. Frieboes, Joseph Chen, Adrianna Masters, Brian J. Williams, Kavitha Yaddanapudi

**Affiliations:** 1 Department of Neurosurgery, University of Louisville, Louisville, KY 40202, USA; jamie.seo@louisville.edu (S.J.S.); brian.williams.3@louisville.edu (B.J.W.); 2 Brown Cancer Center, University of Louisville, Louisville, KY 40202, USA; donald.miller@louisville.edu (D.M.M.); hermann.frieboes@louisville.edu (H.B.F.); adrianna.masters@louisville.edu (A.M.); 3 Department of Bioinformatics, University of Louisville, Louisville, KY 40202, USA; jonah.daneshmand@louisville.edu; 4 Departments of Pathology and Laboratory Medicine, University of Louisville, Louisville, KY 40202, USA; norman.lehman@louisville.edu; 5 Department of Medicine, University of Louisville, Louisville, KY 40202, USA; 6 Department of Bioengineering, University of Louisville, Louisville, KY 40202, USA; dylan.goodin@louisville.edu (D.A.G.); joseph.chen@louisville.edu (J.C.); 7 Department of Radiation Oncology, University of Louisville, Louisville, KY 40202, USA; 8 Department of Microbiology and Immunology, University of Louisville, Louisville, KY 40202, USA; 9 Department of Surgery, University of Louisville, Louisville, KY 40202, USA

**Keywords:** glioblastoma, microglia, recurrence, macrophages, fatty acid

## Abstract

Background: Glioblastoma (GBM) tumors are rich in tumor-associated microglia/macrophages. Changes associated with treatment in this specific cell population are poorly understood. Therefore, we studied changes in gene expression of tumor-associated microglia/macrophages (Iba1+) cells in de novo versus recurrent GBMs. Methods: NanoString GeoMx^®^ Digital Spatial Transcriptomic Profiling of microglia/macrophages (Iba1+) and glial cells (Gfap+) cells identified on tumor sections was performed on paired de novo and recurrent samples obtained from three IDH-wildtype GBM patients. The impact of differentially expressed genes on patient survival was evaluated using publicly available data. Results: Unsupervised analyses of the NanoString GeoMx^®^ Digital Spatial Profiling data revealed clustering based on the transcriptomic data from Iba1+ and Gfap+ cells. As expected, conventional differential gene expression and enrichment analyses revealed upregulation of immune-function-related genes in Iba1+ cells compared to Gfap+ cells. A focused differential gene expression analysis revealed upregulation of phagocytosis and fatty acid/lipid metabolism genes in Iba1+ cells in recurrent GBM samples compared to de novo GBM samples. Importantly, of these genes, the lipid metabolism gene *PLD3* consistently correlated with survival in multiple different publicly available datasets. Conclusion: Tumor-associated microglia/macrophages in recurrent GBM overexpress genes involved in fatty acid/lipid metabolism. Further investigation is needed to fully delineate the role of PLD phospholipases in GBM progression.

## 1. Introduction

Glioblastoma (GBM) is the most common primary malignant brain tumor [1]. Survival is poor, with a median life expectancy of approximately 14 months despite aggressive treatment with surgery, radiotherapy, and chemotherapy [1,2]. The mortality from GBM remains high, with approximately 12,000 cases diagnosed each year and 12,000 deaths occurring each year [2]. Current treatment includes maximally safe surgical excision, radiation, and chemotherapy (Temozolomide); despite these measures, recurrence is inevitable [3]. Clinical trials designed to improve the current standard of care have not been successful in improving treatment results [4]. After disease recurrence, treatment is even less effective, with a median survival of approximately 6 months with aggressive treatment [5,6]. Although immunotherapy, including immune checkpoint inhibitors (e.g., anti-PD-1 antibody Pembrolizumab), has dramatically improved survival in melanoma and non-small-cell lung cancer patients [7], it has not had a significant impact on the survival of GBM patients (either as a replacement for Temozolomide with initial therapy or at the time of recurrence versus Bevacizumab) [8,9]. 

The overall lack of progress in GBM treatment is multifactorial and most likely reflects unique impediments to drug delivery to brain tumors, including the blood–brain barrier limiting the efficacy of cytotoxic therapies and the immune-suppressive tumor microenvironment limiting the efficacy of immunotherapy [8,9,10,11,12,13]. The complex biology of GBM includes dramatic inter-tumoral genetic heterogeneity (tumor differences from patient to patient) as well as intra-tumoral heterogeneity (varied histological appearance and expression pattern in different areas of the same tumor) [14]. The poor prognosis of GBM patients and the lack of progress in clinical trials underscore the critical need for a better understanding of the underlying biology of GBM.

To address the concerns of inter-tumoral molecular heterogeneity, in this pilot study, we identified three patients with paired samples from initial resection at the time of diagnosis and recurrence after treatment and used NanoString GeoMx^®^ Digital Spatial Profiling (DSP) technology to map gene expression changes in de novo versus recurrent GBM tumors resected from these three patients. DSP is a platform for high-plex spatial and temporal profiling of proteins or RNAs, suitable for use on formalin-fixed, paraffin-embedded (FFPE) tissue samples [15,16]. In this pilot study, we show that tumor-associated macrophages/microglia in recurrent GBM overexpress genes involved in fatty acid/lipid metabolism.

## 2. Materials and Methods

### 2.1. Patient Samples

Human tumor tissue samples were surgically resected from patients with glioblastoma (GBM) in the supratentorial compartment of the brain who underwent surgery at the University of Louisville. Both histopathological criteria as well as absence of an *IDH1/2* mutation were used to diagnosed GBM. For Digital Spatial Profiling (DSP) analyses, three patients who previously had surgery for de novo GBM and then recurrent GBM performed at the University of Louisville were identified through a medical records search. All patients provided informed consent to a protocol that was approved by the University of Louisville IRB (IRB# 22.0332 and 20.1107). All specimens were reviewed by a certified neuropathologist to confirm the diagnosis. For each specimen, unstained slides were cut at 5 µm thickness from formalin-fixed, paraffin-embedded (FFPE) blocks and collected on adhesive glass slides. Each of the slides contained a paired primary and recurrent GBM section from each patient. Characteristics of patients included in the DSP analyses are listed in Table 1.

### 2.2. Digital Spatial Profiling—Selection and Segmenting of Regions of Interest

**Figure 1:**
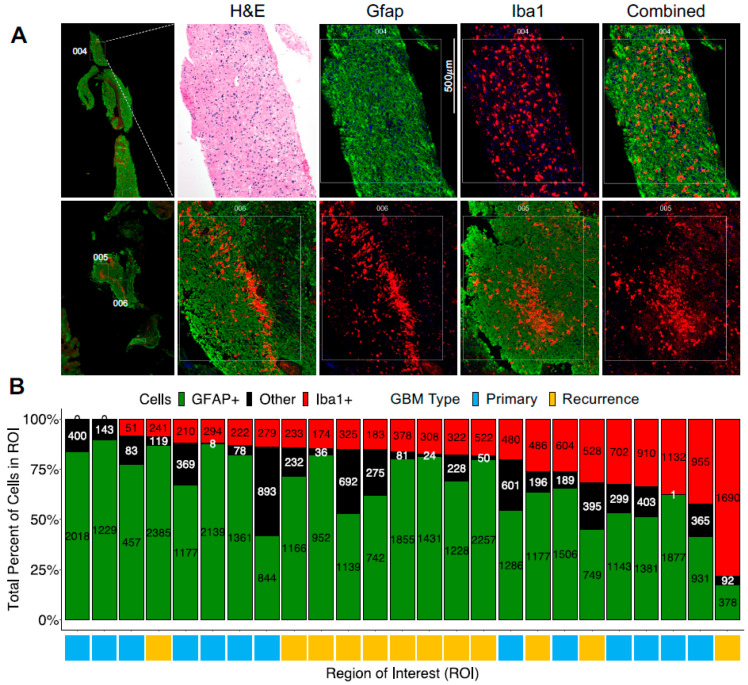
Regions of interest in paired primary and recurrent GBM tissues. (**A**) Photomicrographs of a selected case (Slide 1) of paired primary and recurrent GBM tissues. Close-ups of regions 004 (top row), 005 (bottom row), and 006 (bottom row) are shown. The sections were stained with the visualization markers Iba1 (red), Gfap (green), and DNA (blue). (**B**) Distribution of Gfap+, Iba1+, and other cells within each region of interest. The regions in columns are labeled by GBM type (Primary or Recurrence).

The GeoMx™ instrument and associated commercial software were used to analyze the GBM samples. The FFPE GBM sections were stained with a cocktail of fluorophore-tagged human antibodies against Iba1+, Gfap+, and DAPI, each of which is conjugated with a synthetic oligonucleotide via a UV-labile cross-linker. The stained slides were scanned using the GeoMx^TM^ instrument, and 3-channel immunofluorescent (IF) digital images (Figure 1) were generated to visualize nuclei (DAPI), glial cells (Gfap+), and microglia/macrophages (Iba1+). These images at 20× magnification were then used to select twenty-five rectangular regions of interest (ROIs) across all of the slides, balancing a nearly equal number of ROIs in each tissue. The size of the ROI was the same. The ROIs were selected to capture glia-enriched and microglia/microphage-enriched regions. Two antibody-guided immunofluorescence masks/areas were computer-generated within each ROI to generate distinct segmentation areas to guide laser illumination: the Iba1+ and Gfap+ masks display the highly enriched segments in these cells. The ROIs were then selectively illuminated with cycles of UV light. With each illumination cycle, photocleaved oligos were collected and hybridized for analysis on the NanoString nCounter system.

### 2.3. Data Analysis

#### 2.3.1. Nanostring Data Normalization

Oligonucleotides in each segment (Iba1+ and Gfap+) were quantified with the conventional NanoString nCounter technology. NanoString’s bioinformatics pipeline processed and normalized the data. The normalized data were used for downstream bioinformatic analyses. 

R software version 4.2 (R Foundation for Statistical Computing, Vienna, Austria) and accompanying statistical R packages were used to conduct all analyses and generate plots. Between-group differences in continuous variables were tested using nonparametric *t* tests. Significance was set at a two-tailed *p*-value of 0.05 unless stated otherwise below.

#### 2.3.2. Unsupervised Clustering

Hierarchical clustering was performed using the “ward.D” method. Dimensionality reduction of the expression data with *t-distributed* Stochastic Neighbor Embedding (t-SNE) was performed to facilitate visualization using the following parameters (dimensions = 2, perplexity = 15, iterations = 1000) using the Rtsne package (ver: 0.16). 

#### 2.3.3. Differential Expression and Pathway Analyses

Differential expression was performed using DESeq2 (v: 1.38.3). Any gene with a log fold change greater than ±1 and a *p*-value < 0.05/total number genes (i.e., Bonferroni’s correction) was considered to be differentially expressed. Pathway analyses were conducted on these differentially expressed genes using clusterProfiler (v: 3.17).

#### 2.3.4. Survival Analyses 

Survival data from IDH wildtype GBM samples available in GlioVis (http://gliovis.bioinfo.cnio.es; accessed on 1 August 2023) were downloaded. Because each dataset analyzed the expression data differently, we performed survival analysis by first identifying a cut point in the range of the normalized expression level of the gene of interest that was associated with the greatest divergence in survival. This cut point was used to dichotomize the dataset into 2 groups (i.e., patients with high and low expression of the gene of interest). For visualization, we plotted the survival data of the 2 groups using right-censored Kaplan–Meier curves. We used the survminer v0.4.9 R package for this analysis and plot generation. We compared the curves using the log-rank statistical test.

## 3. Results

We analyzed microglia/macrophages in newly diagnosed and recurrent matched pairs of GBM (IDH-wildtype) samples from three patients aged 43, 62, and 68 years. Inclusion criteria included diagnosis of GBM from initial resection, lack of isocitrate dehydrogenase mutation (IDH), and additional resection stored in the biorepository after recurrence. One was female, and all were non-Hispanic Caucasians who received the standard-of-care treatment with radiation and Temozolomide. The patients had overall survival of 148, 336, and 564 days. Variables including age, integrated molecular diagnosis, treatments, and survival are provided in Table 1.

### 3.1. The Presence of a Tumor-Associated Microglia/Macrophage Niche in GBM

Most of the 25 selected ROIs were enriched with Gfap+ cells, except for 1, where Iba1+ cells constituted nearly 75% of the cells. Two ROIs did not have any Iba1+ cells. Rarely were the Iba1+ cells diffusely located (Figure 1A, top panel); instead, in many ROIs, the Iba1+ cells formed a niche (i.e., they were spatially together with high local density; Figure 1A, bottom panels).

### 3.2. Digital Spatial Transcriptomic Profiling of Tumor-Associated Microglia/Macrophages

**Figure 2:**
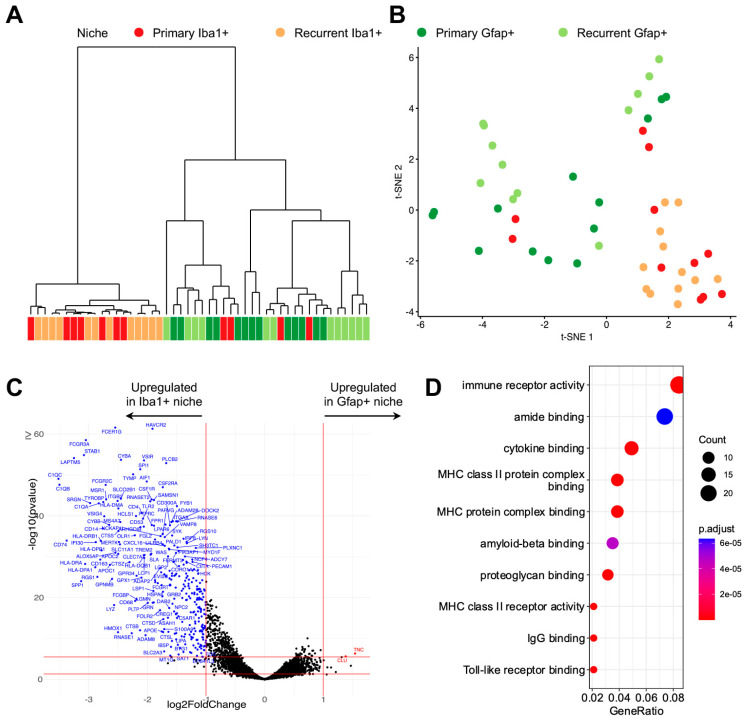
Unsupervised clustering of all regions and differential expression analysis of Iba+ niche versus Gfap+ niche. (**A**) Hierarchical clustering dendrogram of all regions, color-labeled by niche. (**B**) t-SNE projection of all regions, color-labeled by niche. In both (**A**,**B**), a clear separation can be seen between Iba+ and Gfap+ niches. (**C**) A volcano plot showing differentially expressed genes between Iba+ and Gfap+ niches. Each dot represents a gene, with red dots indicating genes that are significantly upregulated in recurrent tumors (log2 fold change > 1 and adjusted *p*-value < 0.05), blue dots indicating genes that are significantly downregulated (log2 fold change < −1 and adjusted *p*-value < 0.05), and black dots indicating genes that are not significantly differentially expressed. The red lines represent the threshold for statistical significance (adjusted *p*-value < 0.05), and the vertical red lines represent the threshold for fold change (log2 fold change > 1 and < −1). Gene symbols for the most significantly differentially expressed genes are shown. (**D**) Dot plot showing Gene Ontology enriched terms of differentially expressed genes (DEGs) between Iba+ and Gfap+ niches. The terms shown suggest an immune-related activity, which is to be expected within Iba+ niches.

We conducted a digital spatial transcriptomic profiling of the Gfap+ and Iba1+ segments within the 25 different ROIs from matched primary and recurrent GBM samples of these three patients (Figure 1A). The transcriptomic profiling was successfully conducted on varying numbers of Gfap+ and Iba+ nuclei (DAPI positive), with a minimum of 51 nuclei (Figure 1B). Unsupervised hierarchical clustering of the gene expression data from digital spatial capturing of Iba1+ and Gfap+ cells revealed identity-specific clustering (Figure 2A). Four Iba1+ segments in the ROIs of newly diagnosed GBMs clustered with Gfap+ segments. To confirm this finding with another unsupervised form of analysis, we reduced the high-dimensional gene expression data to two dimensions using the t-SNE algorithm. The resulting two-dimensional plot of the segments revealed local clustering of Iba1+ segments, confirming the prior results (Figure 2B). In both analyses, the segments did not cluster based on the GBM’s recurrence status. We then identified differentially expressed genes. Nearly all of these genes were upregulated and specific to Iba1+ cells (Figure 2C). The KEGG pathway analysis of these genes revealed that most of them contributed to immune functions (Figure 2D).

### 3.3. Differentially Expressed Microglial/Macrophage Genes in Recurrent GBM

**Figure 3:**
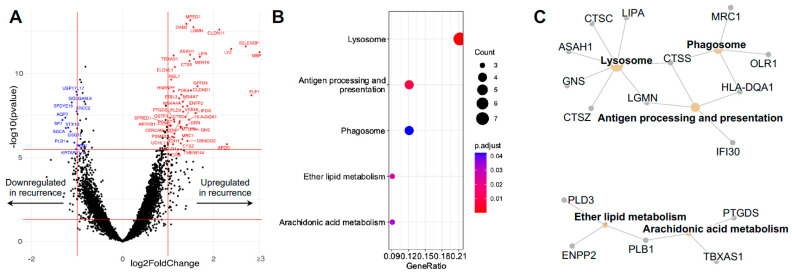
Differential expression analysis of primary versus recurrence regions within the Iba+ niche. (**A**) Volcano plot of differential gene expression in Iba1+ microglia/macrophages in recurrent GBM tissues compared to primary GBM tissues (all 25 ROIs from *n* = 3 patients). Each dot represents a gene, with red dots indicating genes that are significantly upregulated in recurrent tumors (log2 fold change > 1 and adjusted *p*-value < 0.05), blue dots indicating genes that are significantly downregulated (log2 fold change < −1 and adjusted *p*-value < 0.05), and black dots indicating genes that are not significantly differentially expressed. The dashed red lines represent the threshold for statistical significance (adjusted *p*-value < 0.05), and the vertical red lines represent the threshold for fold change (log2 fold change >1 and <−1). Gene symbols for the most significantly differentially expressed genes are shown. (**B**) Dot plot showing Gene Ontology enriched terms of DEGs between primary and recurrent GBMs within the Iba+ niche. (**C**) Network plot of the Gene Ontology enriched terms and associated represented DEGs.

To understand differences in the Iba1+ population in newly diagnosed and recurrent GBMs, we examined the differentially expressed genes. Most of the differentially expressed genes were upregulated in the Iba1+ cells present in the recurrent GBM samples (Figure 3A). The KEGG pathway analysis of these genes revealed that they belonged to two general biological processes: phagocytosis and fatty acid/lipid metabolism (Figure 3B). Phagocytosis incites the lysosome and antigen processing and presentation. The differentially expressed genes relevant to phagocytosis are (depicted in Figure 3C) MRC1, HLA-DQA1, OLR1, LIPA, CTSC, ASAH1, GNS, CTSZ, LGMN, and IFI30; on the other hand, PLD3, ENPP2, PLB1, TBXAS1, and PTGDS were identified as upregulated genes in recurrent GBMs that are involved in fatty acid/lipid metabolism. Differentially expressed genes in the Gfap+ cells could not be readily attributed to any specific biological process using the KEGG pathway analysis.

### 3.4. Lipid Metabolism Gene PLD3 Correlates with Survival in Several GBM Cohorts

**Figure 4:**
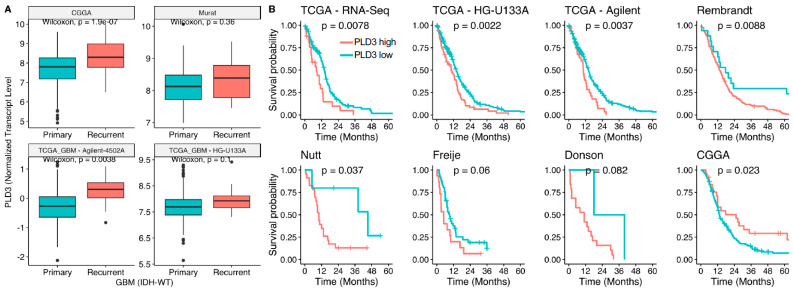
Changes in the level of expression of PLD3 associated with GBM recurrence and its correlation with survival using publicly available datasets. (**A**) Box plots showing Wilcoxon test comparison of PLD3 expression between primary and recurrent GBMs across four publicly available datasets. (**B**) Kaplan–Meier plots showing survival analysis between PLD3-high and PLD3-low patients across the eight publicly available datasets.

While prior works have shown the prognostic value of genes, such as IFI30, responsible for phagocytosis-mediated protein degradation [17], here, we evaluated whether lipid metabolism genes correlated with survival using the publicly available datasets. Of the five upregulated genes, PLD3 consistently correlated with survival in nearly all cohorts tested. Analysis of publicly available transcriptomic data comparing newly diagnosed and recurrent GBMs revealed that levels of PLD3 were higher in recurrent GBMs in all four cohorts analyzed (Figure 4A). Importantly, higher levels of PLD3 correlated with lower survival in newly diagnosed GBM patients (IDH-wildtype without CpG island methylator phenotype) in nearly all publicly available GBM cohort data (Figure 4B). 

## 4. Discussion

In this small proof-of-concept study, we performed gene expression profiling of paired de novo and recurrent GBM (from 25 different ROIs in tissues from n = 3 patients) using DSP with an overarching goal of identifying tumor-associated microglia/macrophage-intrinsic molecular aberrations within the GBM lesions that may be responsible for disease relapse. Our main finding is the identification of genes upregulated in the Iba1+ cells in the recurrent GBM samples that primarily belong to two general biological processes: phagocytosis and fatty acid/lipid metabolism. Importantly, of these genes, the increased expression of the lipid metabolism gene *PLD3* consistently correlated with survival in several GBM cohorts. Overall, this study highlights the utility of snapshot, spatial multiomic approaches to gain insight into GBM recurrence and its molecular complexities [18].

The DSP platform (NanoString Technologies) relies on the multiplexed readout of proteins or RNAs using oligonucleotide tags; oligonucleotide tags are attached to affinity reagents (antibodies or RNA probes) through a photocleavable (PC) linker. Photocleaving light is projected onto the tissue sample to release PC oligonucleotides in any spatial pattern across a region of interest (ROI) covering 1 to ~5000 cells. With each illumination cycle, photocleaved oligos are collected and hybridized for analysis on the NanoString nCounter system. DSP is capable of high sensitivity within an ROI, with RNA detection feasible down to ~600 individual mRNA transcripts, allowing for in-depth analysis of prognostic and predictive biomarkers in a cell-type-specific manner. 

GBM tumors display several layers of immune and molecular heterogeneity, including variations in architectural tumor arrangements and spatial infiltration of cells of the tumor immune microenvironment, which includes microglia/macrophages [19]. This heterogeneity, which can be both dynamic and temporal, is apparent not only among different individuals but also in different regions of the same tumor. To delineate this heterogeneity between primary and recurrent GBM, we have profiled gene expression in glioma-rich and microglia/macrophage-rich spatially defined ROIs using Gfap and Iba1 as the respective visualizing markers. Gfap is the most common glial biomarker, and it is expressed by both normal and malignant glial cells [20]. Iba1-expressing tumor-associated microglia/macrophages are recruited to the tumors and constitute a single cluster in the tumor microenvironment. However, studies have identified brain-resident microglia cells as an ontogenically distinct population from the peripheral macrophages; they are long-lived with self-renewal capability without relying on bone-marrow-derived progenitors [21,22,23,24,25,26]. Microglia, under homeostatic conditions, are initiators of the innate immune response; however, under the influence of tumor-generated signals, they become immunosuppressive, tumor-promoting cells, and they are associated with worse prognosis in GBM [27,28,29]. Some contributions of glioma-associated microglia toward tumor progression are well-recognized and documented [30,31,32,33], while many others are yet to be discovered, especially those that contribute to disease recurrence. In this report, two such potential pathways are identified. Under homeostatic conditions, microglia have a transcriptional signature consisting of diminished expression of MHC Class II molecules and lipid metabolism genes and enriched in *P2RY13*, *TMEM119*, *CX3CR1*, *P2RY12*, *CSF1R*, *MARCKS*, and *SELPLG* genes [28,34]. Our study provides new insights into variations in the composition of tumor-resident microglia/macrophages present in de novo and recurrent GBM; those in recurrent GBMs have a higher expression of genes related to phagocytosis and lipid metabolism, suggesting a phenotypic shift towards a more inflammatory and pro-tumoral phenotype. Our findings of microglia/macrophage-specific gene expression changes in recurrent GBM may also indicate their potential role in response to therapeutic intervention.

To further examine the relationship between spatial microenvironmental features and disease outcome, we evaluated microglial/macrophage gene signatures defined by the DSP analysis and their association with clinical outcomes in GBM. We found that higher levels of *PLD3* correlated with lower survival in newly diagnosed GBM patients. The *PLD3* gene encodes a member of the phospholipase D (PLD) family of enzymes that catalyzes the hydrolysis of membrane phospholipids and generates bioactive lipid mediator phosphatidic acid [35]. PLD activity has been shown to promote cancer progression, including growth, metabolism, and mobility [36]. An increase in global PLD activity was shown to induce matrix metalloproteinase 2 (MMP2) secretion and glioma cell invasion [37]. In our study, microglia/macrophage cell-type-specific increase in *PLD3* expression in recurrent GBM suggests that dysregulation in the phospholipase signaling circuit and lipid-mediated activation of downstream effectors, specifically in tumor-associated microglia/macrophages, can contribute to tumor recurrence. Further investigation is needed to fully delineate the role of PLD phospholipases in the context of de novo and recurrent GBM.

Recent advancements in our comprehension of cancer metabolism have unveiled the highly heterogeneous nature of GBM metabolism [38,39,40]. Cancer stem cells as well as stromal cells within GBM display distinct metabolic characteristics that underpin their unique functionalities. GBM microenvironment is lipid-rich and fatty acid metabolism has emerged as a significant focus in this area, given its critical role in numerous biological processes that are central to GBM pathogenesis. Lipid metabolism influences several processes that play a crucial role in the development and progression of GBM. Tumor-mediated reprogramming of these processes encompasses changes in import/export signaling pathways, lipid anabolism, catabolism (fatty acid oxidation), regulation of ferroptosis, and alteration in de novo lipid synthesis and signaling pathways that include lipogenesis and cholesterol synthesis and targeting these pathways may represent a potential therapeutic strategy to overcome GBM progression and disease recurrence [41,42,43]. Fatty acid metabolism has been shown to contribute to immune suppression in GBM. Published work has demonstrated that treatment with etomoxir, an inhibitor of fatty acid oxidation, suppressed myeloid-derived suppressor cell metabolism and their T cell immunosuppressive functions in tumors [44]. Furthermore, immune suppressive T regulatory cells (T_regs_) in GBM have been shown to express elevated levels of multiple fatty acid transporters and inhibition of fatty acid transporters as well as fatty acid metabolism in T_regs_ resulted in inhibition of their suppressive activity [45,46]. These studies suggest that fatty acid metabolism can influence the immune suppressive activity of tumor-infiltrating myeloid cells in GBM. However, numerous questions remain unanswered in the field of fatty acid metabolism in GBM. Efforts are warranted to unravel these intricate processes. 

Last, the influence of the immune microenvironment on the GBM subtype and evolution is well-established [47,48]. Recent studies have also revealed its influence on the metabolome [49,50,51]. With our pilot data, we, too, show the importance of immune-cell-specific ASAH1 in recurrent GBM, as did a recent 2023 proteometabolomics study [49].

A limitation of this study is the utilization of samples from only three patients available for the spatial profiling; therefore, further validation of results using a larger patient cohort with a greater number of ROIs is required. Other limitations include the absence of validation of the gene expression data at the protein level, lack of single-cell expression data, and limited information on the molecular pathways activated in other myeloid and non-myeloid immune cells present in the tumor stromal regions selected. Despite these shortcomings, the data in this pilot study suggest that the tumor microenvironment of microglia/macrophage-rich regions in recurrent GBM may hold prognostic value for overall patient survival with therapeutic implications, and this study opens further investigations into confirmation and the biological basis/mechanism of our findings.

## Figures and Tables

**Table 1:** Characteristics of three patients with glioblastoma (IDH1 wildtype).

Patient Study ID	MGMT Promoter Status	Gender	Age at Diagnosis	Collection Date (Days Relevant to Diagnosis)	Disease Status at Collection	Treatment	RT End Date (Days Relevant to Collection)	Overall Survival (Days)
CDSR-01276-CNS	Unmethylated	Male	43	0	De Novo	Resection/RT/ Temozolomide		336
CDSR-01276-CNS		Male		206	Progression	Reresection	151	
CDSR-01396-CNS	Methylated	Female	62	0	De Novo	Resection/RT/ Temozolomide		564
CDSR-01396-CNS		Female		358	Progression	Needle Biopsy	36	
CDSR-01595-CNS	Unmethylated	Male	68	0	De Novo	Resection/ Lomustine		148
CDSR-01595-CNS		Male		77	Progression	Reresection	N/A	

## Data Availability

The datasets generated and analyzed in this study are available from the corresponding author upon reasonable request.
